# Caudodorsal approach combined with in situ split for laparoscopic right posterior sectionectomy

**DOI:** 10.1007/s00464-022-09657-1

**Published:** 2022-10-06

**Authors:** Chongwei Yang, Rixin Zhang, Ling Zhu, Xiaolin Zheng, Kai Li, Pi-Xiao Wang

**Affiliations:** Department of Hepatobiliary Surgery, The Central Hospital of Wuhan, Tongji Medical College, Huazhong University of Science and Technology, Wuhan, 430014 China

**Keywords:** Laparoscopic hepatectomy, Right posterior section, Dorsal approach, In situ

## Abstract

**Background:**

Laparoscopic right posterior sectionectomy (LRPS) was technically challenging and lack of standardization. There were some approaches for LRPS, such as caudal approach and dorsal approach. During our practice, we initiated pure LRPS using the caudodorsal approach with in situ split and present several advantages of this method.

**Methods:**

From April 2018 to December 2021, consecutive patients who underwent pure LRPS using the caudodorsal approach with in situ split at our institution entered into this retrospective study. The key point of the caudodorsal approach was that the right hepatic vein was exposed from peripheral branches toward the root and the parenchyma was transected from the dorsal side to ventral side. Specially, the right perihepatic ligaments were not divided to keep the right liver in situ before parenchymal dissection for each case.

**Results:**

11 patients underwent pure LRPS using the caudodorsal approach with in situ split. There were 9 hepatocellular carcinoma, 1 sarcomatoid hepatocellular carcinoma, and 1 hepatic hemangioma. Five patients had mild cirrhosis and 1 had moderate cirrhosis. All the procedures were successfully completed laparoscopically. The median operative time was 375 min (range of 290–505 min) and the median blood loss was 300 ml (range of 100–1000 ml). Five patients received perioperative blood transfusion, of which 1 patient received autologous blood transfusion and 2 patients received blood transfusion due to preoperative moderate anemia. No procedure was converted to open surgery. Two patients who suffered from postoperative complications, improved after conservative treatments. The median postoperative stay was 11 days (range of 7–25 days). No postoperative bleeding, hepatic failure, and mortality occurred.

**Conclusion:**

The preliminary clinical effect of the caudodorsal approach with in situ split for LRPS was satisfactory. Our method was feasible and expected to provide ideas for the standardization of LRPS. Further researches are required due to some limitations of this study.

**Supplementary Information:**

The online version contains supplementary material available at 10.1007/s00464-022-09657-1.

There was almost no restricted area for laparoscopic hepatectomy at present. But laparoscopic anatomical hepatectomy, especially for difficult areas, such as the right posterior section, still increase the score in the main difficulty scoring systems [1–3]. Utilizing the unique laparoscopic caudodorsal view, Dorsal approach, firstly reported in laparoscopic left hemihepatectomy in 2014 [4], was expected to be feasible and provide an advantage for laparoscopic right posterior sectionectomy (LRPS) [5]. During our practice, we initiated pure LRPS using the caudodorsal approach with in situ split. Here, we present our results in patients who underwent pure LRPS using this method at our institution and described our recent standardized procedure.

## Materials and methods

### Patients

From April 2018 to December 2021, consecutive patients who underwent pure LRPS using the caudodorsal approach with in situ split at our institution entered into this retrospective study. This study was approved by the Ethics Committee of the Central Hospital of Wuhan, Tongji Medical College, Huazhong University of Science and Technology (Wuhan, China, approval no. WHZXKYL2022-080).

### Surgical procedures

**Fig. 1:**
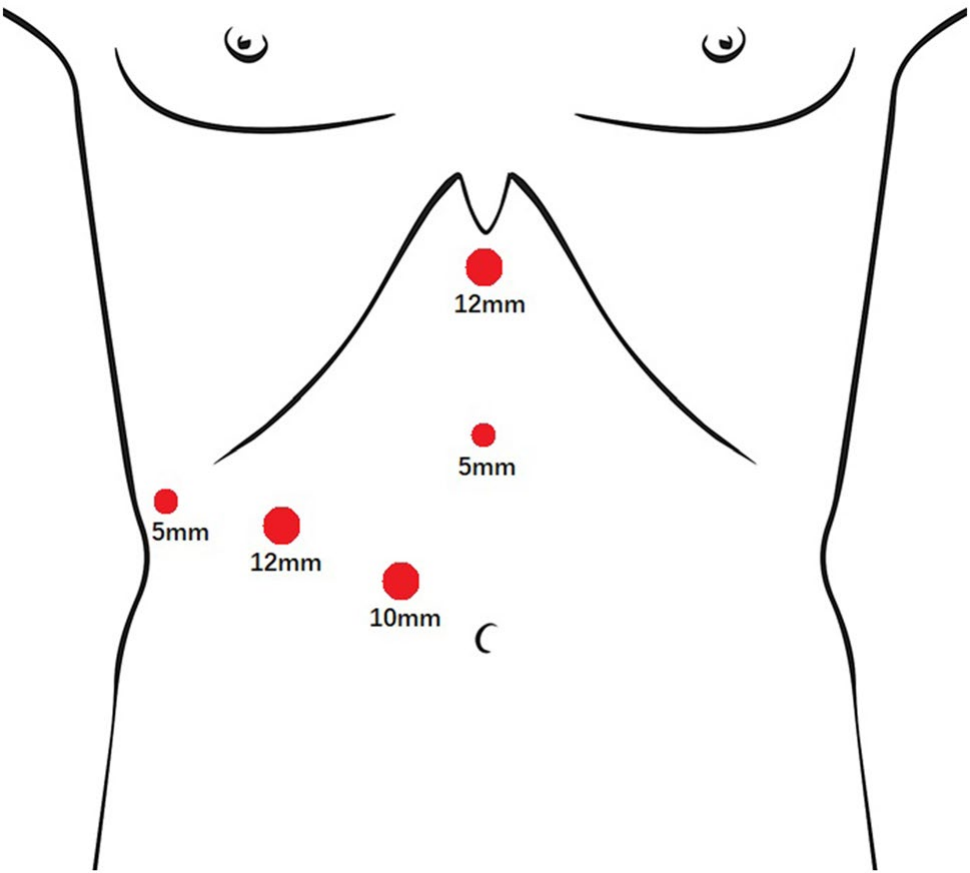
Trocar position

The patient was placed in a supine position, with approximately 30-degrees of right-sided raised. Trocar position was shown in Fig. 1. A pneumoperitoneum was established through a 10-mm right pararectal trocar, and maintained with carbon dioxide at 12–14 mmHg. Two 12-mm trocars were located in the right midclavicular line and inferior to the xiphoid process, and a further two 5-mm trocars were located in the rightmost of the costal margin and the supra-umbilical region. The main surgeon stood on the patient’s right side.

**Fig. 2:**
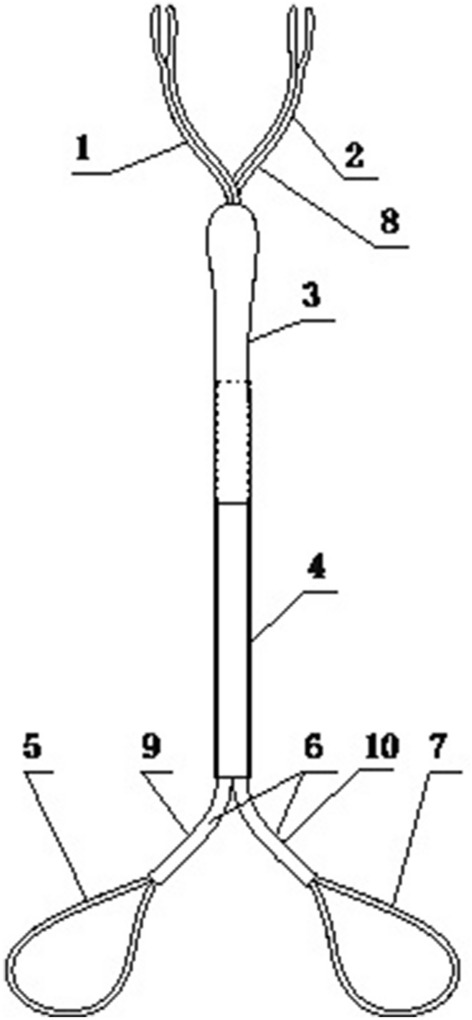
Diagram of “Y”-shaped vascular occlusion device (engineering drawing): 1—the first blocking rope for hilar clamping, 2—the second blocking rope for infrahepatic inferior vena cava clamping, 3—the silicone bolster, 4—the hard tube, 5—the first blocking ring, 6—the elastic silicone cathead, 7—the second blocking ring, 8—the operating side, 9—the elastic silicone cathead for first blocking, 10—the elastic silicone cathead for second blocking

**Fig. 3:**
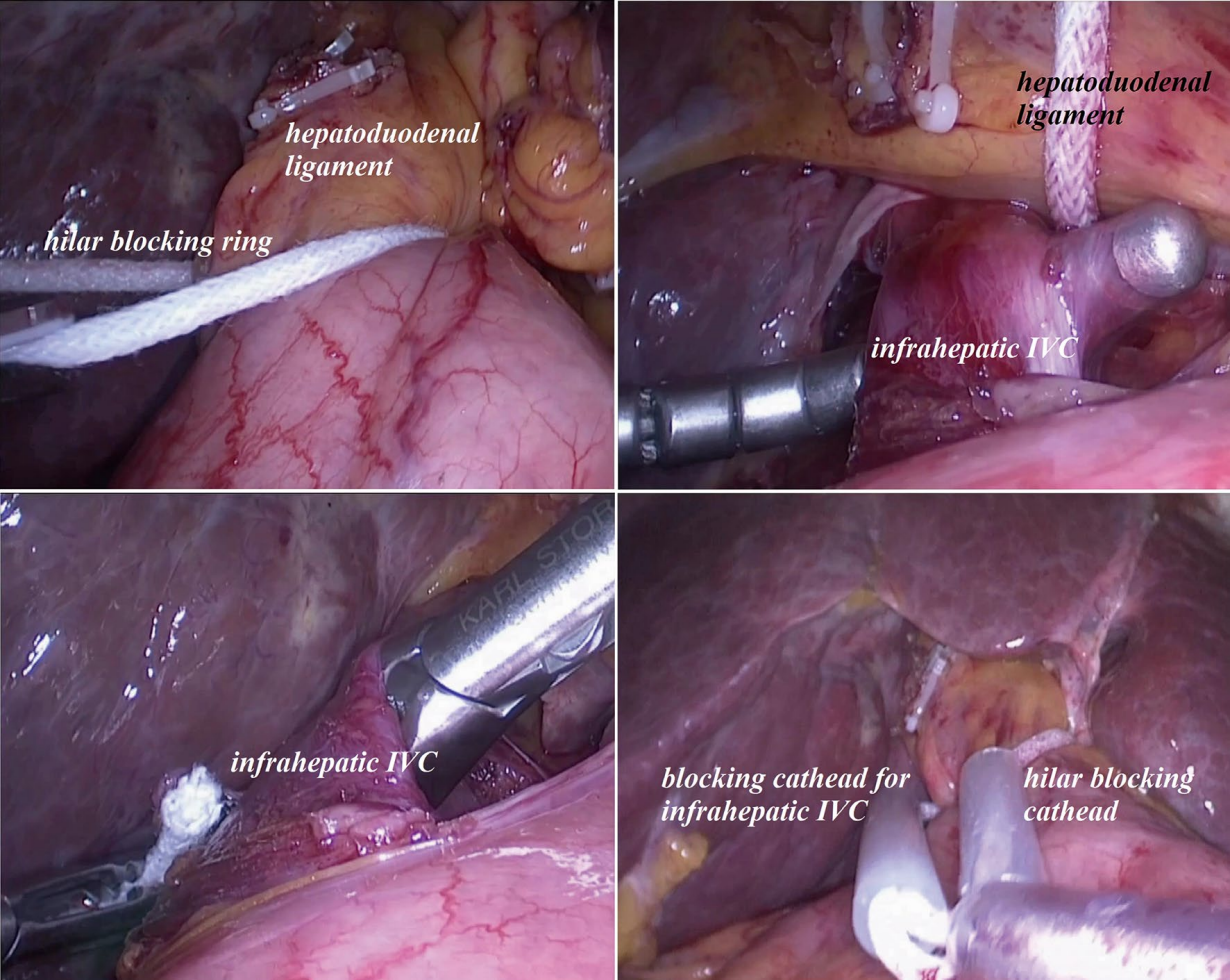
“Y”-shaped vascular occlusion device under the laparoscopic view

Either Pringle maneuver or Pringle maneuver combined with infrahepatic inferior vena cava (IVC) clamping was used to control blood loss if necessary. The Pringle maneuver was a common method in laparoscopic hepatectomy, using cycles of clamp/unclamp intervals of 15/5 min. Infrahepatic IVC clamping could reduce blood loss during liver resection by decreasing the central venous pressure [6]. As for safety, partial clamping of the infrahepatic IVC was performed to maintain hemodynamic stability and avoid vein thrombosis. We developed a device, named as “Y”-shaped vascular occlusion device for laparoscopic hepatectomy, by combining Pringle maneuver with infrahepatic IVC clamping to control bleeding (Fig. 2). The “Y”-shaped vascular occlusion device was preset before parenchymal transection (Fig. 3). Compared with the traditional method, this device could make the operation more convenient, and less damage. Of note the patient’s mean arterial pressure should be maintained above 60 mmHg to avoid acute kidney injury when using infrahepatic IVC clamping [7].

Firstly, the gallbladder was removed, and the ligamentum teres hepatis and falciform ligament of the liver were divided. The right perihepatic ligaments were not divided to keep the right liver in situ. Then, the caudate lobe was dissected along the anterior border of the IVC in the unique laparoscopic caudodorsal magnified view, exposing and transecting the short hepatic veins. With further dissection toward the cranioventral side, the dorsal side of the posterior Glissonean pedicle was exposed. At the Rouviere’s groove, the ventral side of the root of the posterior pedicle was revealed by dissection between the pedicle surface and liver parenchyma. Hung with a tape, the posterior pedicle and its branches were transected using a linear stapler or clips. Notably, the main trunk of the right hepatic vein (RHV) was very close to the pedicle. When the exposure was poor, the liver can be divided to obtain enough space before the liver pedicle was transected to avoid damage to the RHV.

**Fig. 4:**
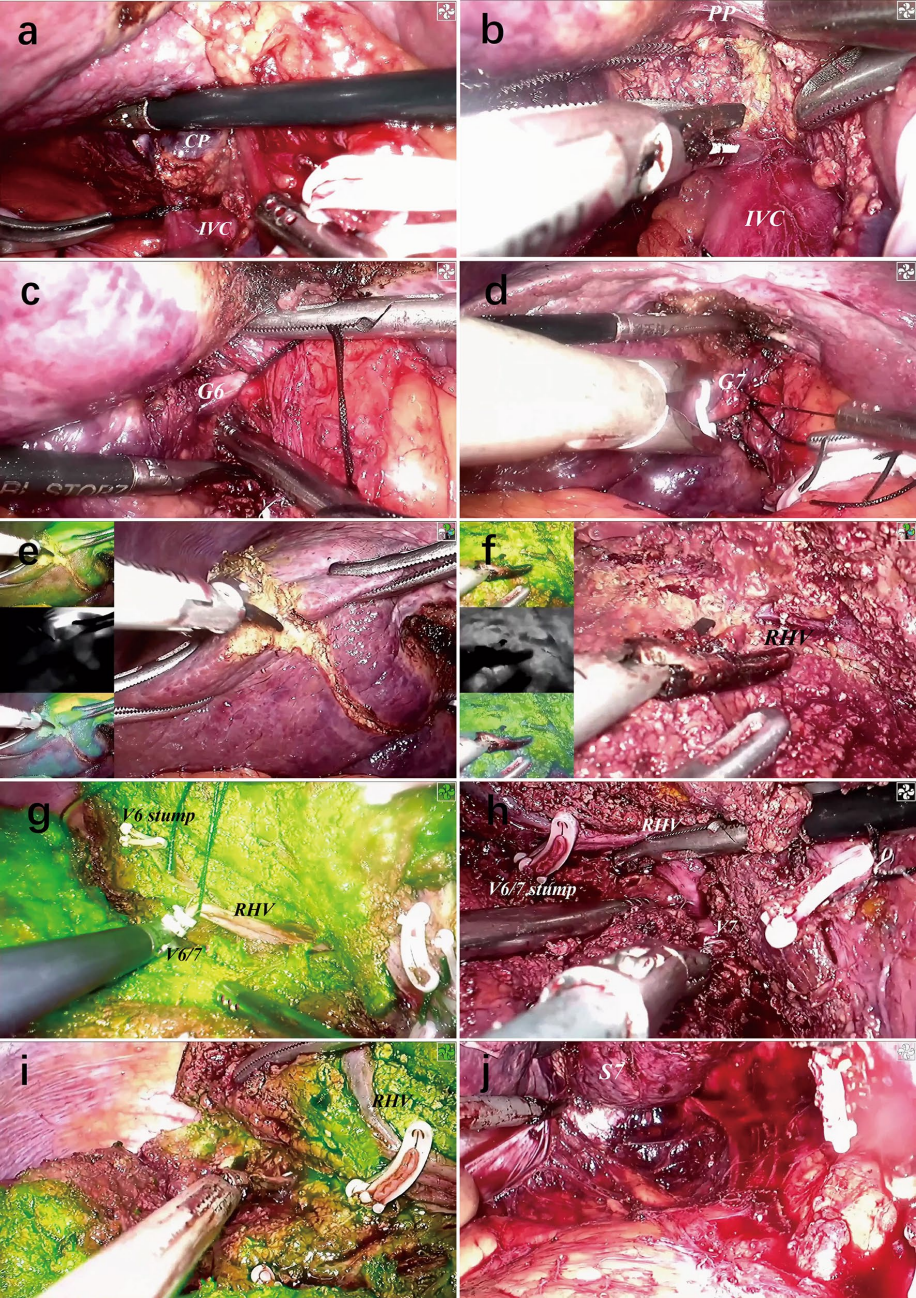
Intraoperative key view during surgery. **a** Isolation of the short hepatic veins by dissecting the caudate lobe, **b** transect the caudate lobe to expose the dorsal side of posterior pedicle, **c** hang segment six pedicle after the dorsal and ventral side were exposed, **d** exposure of segment 7 pedicle after segment 6 pedicle was transected, **e** parenchymal transection of the caudal side with ICG fluorescence imaging, **f** expose the RHV trunk toward its root by dissecting parenchyma at the caudodorsal side, **g** ligate the intersegmental vein, **h** isolate segment seven vein and reveal the whole process of the RHV, **i** transect the cranioventral parenchyma along the plane with ICG fluorescence imaging, **j** divide the perihepatic ligaments at last. *CP* caudate process, *IVC* inferior vena cava, *PP* posterior pedicle, *G6* segment 6 Glissonean pedicle, *G7* segment 7 Glissonean pedicle, *RHV* right hepatic vein, *V6*: segment 6 vein, *V6/7* intersegmental vein between segments 6 and 7, *V7* segment 7 vein, *S7* segment 7, *ICG* indocyanine green

After transecting the hepatic pedicle, the ischemic line could be obtained on the liver surface which was identified as the boundary between the right posterior and anterior sections. Furthermore, laparoscopic ultrasonography and ICG fluorescence imaging were used to obtain a more precise boundary. Liver parenchymal transection of the caudal side along this dissection plane started with the ultrasonic dissector by the main surgeon. To fully expose the plane, the assistant should pull the right anterior section to the ventral and cranial side. Liver transection proceeded to find the peripheral branches of the RHV. Due to the thin parenchyma at the caudodorsal side of the RHV, the main trunk of the RHV could be exposed by dissecting a small amount of liver parenchyma. Continuously dissecting toward the root of the RHV, its whole process was revealed and all the branches draining the right posterior section were ligated and cut off. Indeed, it was also feasible to dissect the liver parenchyma at the cranioventral side after exposing part of the main trunk of the RHV, and then continue to dissect toward the root at the caudodorsal side. Subsequently, the remnant liver parenchyma at the cranioventral side was dissected along the plane guided by the diaphragmatic demarcation and the exposed RHV. Finally, the right perihepatic ligaments was divided to complete resection of the right posterior section without lifting up. The resected specimen was placed in a plastic bag and removed without fragmentation through a suprapubic transverse incision. Intraoperative key view during surgery was shown in Fig. 4.

## Results

Between April 2018 and December 2021, 11 consecutive patients underwent pure LRPS using the caudodorsal approach with in situ split at our institution. The patients’ characteristics are described in Table 1. The median patient age was 67 years (range of 49–73 years), and there were more male than female patients (7/11). Of the 11 patients, 9 had hepatocellular carcinoma, 1 had sarcomatoid hepatocellular carcinoma, and the other 1 had hepatic hemangioma. The median body mass index was 22.1 kg/m^2^ (range of 17.3–30 kg/m^2^). Each patient had preoperative Child–Pugh class A liver function. 5 patients had mild cirrhosis and 1 had moderate cirrhosis. All the procedures were successfully completed laparoscopically. The median operative time was 375 min (range of 290–505 min) and the median blood loss was 300 ml (range of 100–1000 ml). Pringle maneuver was chosen in 7 patients and the other 4 patients were applied with the “Y”-shaped vascular occlusion device. 5 patients received perioperative blood transfusion, of which the 1 with hepatic hemangioma received autologous blood transfusion and 2 patients (case 1, 10) received blood transfusion due to preoperative moderate anemia. No procedure was converted to open surgery. With regard to postoperative complications, one patient suffered from bile leakage and was cured by delayed drainage catheter removal. Another patient, who experienced intraoperative massive blood loss due to moderate cirrhosis, developed abdominal infection on the tenth postoperative day. Fortunately, she was discharged after drug treatment with equally good outcome. The median postoperative stay was 11 days (range of 7–25 days). No postoperative bleeding, hepatic failure, and mortality occurred.

**Table 1:** Patients’ characteristics

Number	Age (year)	BMI (kg/m^2^)	Diagnosis	Cirrhosis	Blood flow control	Operative time (min)	Blood loss (ml)	POS (days)	Postoperative complications
1^a,b^	64	22.7	SHCC	Mild	P	490	350	12	None
2^a^	68	24.2	HCC	None	P	405	400	11	None
3^a,b^	61	17.3	HCC	None	P	360	600	13	None
4^b^	62	30	HCC	Moderate	Y	420	1000	25	Abdominal infection
5^a^	72	25	HCC	Mild	Y	305	150	9	None
6^a^	67	17.3	HCC	Mild	Y	360	200	12	None
7	72	22.6	HCC	None	Y	375	400	12	Bile leakage
8^a^	62	20.8	HCC	None	P	505	300	11	None
9	70	19.5	HCC	Mild	P	320	100	7	None
10^a,b^	73	22.1	HCC	Mild	P	290	200	10	None
11^b^	49	18.5	HM	None	P	420	200	7	None
Median	67	22.1				375	300	11	

*BMI* body mass index, *SHCC* sarcomatoid hepatocellular carcinoma, *HCC* hepatocellular carcinoma, *HM* hepatic hemangioma, *P* pringle maneuver, *Y* “Y”-shaped vascular occlusion device, *POS* postoperative stay

^a^Case 1, 2, 3, 5, 6, 8, 10 were male

^b^Case 1, 3, 4, 10, 11 received perioperative blood transfusion

## Discussion

Laparoscopic liver resection had rapidly evolved during the last decade, and showed encouraging potential in improving outcomes for liver malignancy [8]. Compared with open surgery, LRPS had advantages in operative time, blood loss, and length of hospital stay [9], which had been routinely performed in some large centers. However, it was difficult to effectively expose the visual field and exactly hold the dissection plane for LRPS, due to deep location of the posterior section and multiple variations of the hepatic pedicle [10, 11]. As a result, LRPS was technically challenging and lack of standardization. Some surgeons placed patients in the left lateral position to obtain a clear surgical view, facilitating liver exposure and parenchymal transection [12–14]. As with most, the supine position remained our preferred method for LRPS as well as other laparoscopic hepatectomies. Since 2009, our team had made efforts in improving techniques for anatomical liver segment resection and had formed standardized procedure during practice, especially for some complex liver resections.

Regarding the dissection of the right posterior pedicle, we initially performed extrafascial approach at the Rouviere’s groove, by which the pedicle cannot be completely encircled due to anatomical variations. Instead, the caudate lobe-first approach was adopted to effectively expose the right posterior pedicle and reduce omissions, especially the pedicle of segment seven. In addition, because there was no clear boundary between the right caudate lobe and the right posterior section, initiate excision of the right caudate lobe exposed the right edge of the IVC in advance, which could be a landmark for effectively mastering the dissection plane [5].

**Fig. 5:**
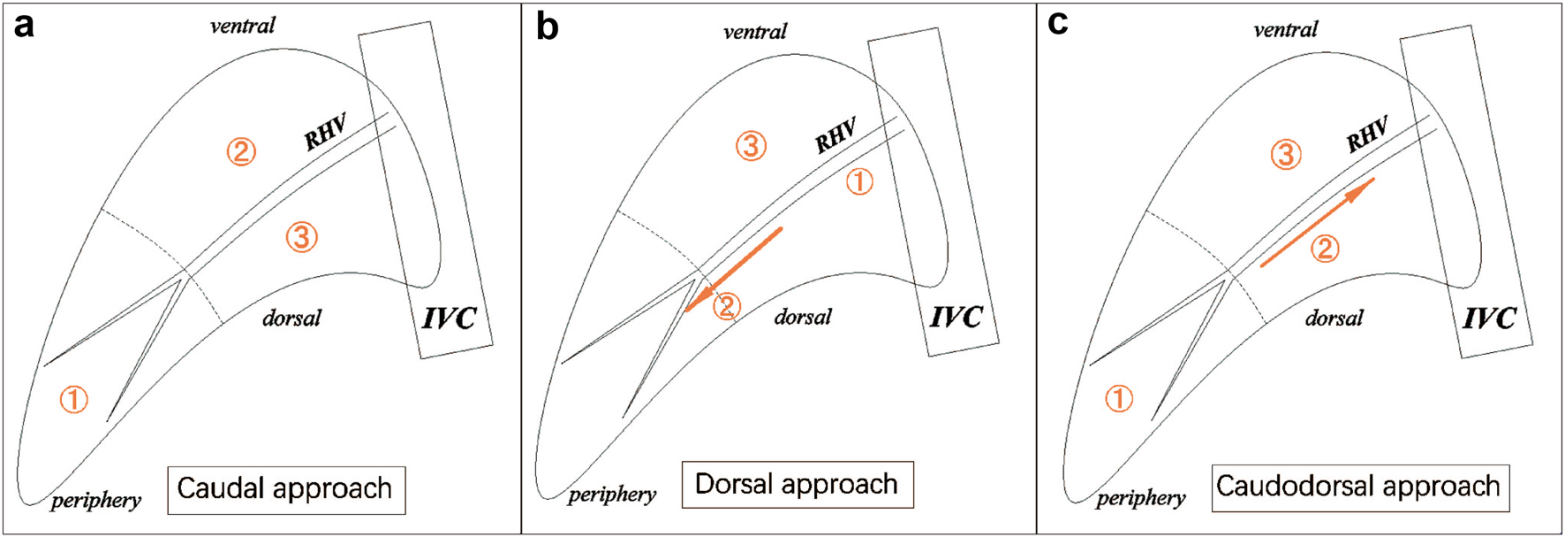
Schematic representation of different approaches for LRPS. **a** Caudal approach, **b** Dorsal approach, **c** Caudodorsal approach. ① ② ③ represents the order of parenchymal dissection and exposure of the RHV. *IVC* Inferior vena cava, *RHV* right hepatic vein

Makuuchi proposed that the intersegmental vein should be exposed and regarded as the border for liver parenchymal transection in anatomic liver resection [15]. The conventional caudal approach was routinely used for anatomic LRPS, which the RHV was exposed from peripheral branches toward its root and the parenchyma was transected from the ventral side to dorsal side (Fig. 5A) [16–18]. Recently, Honda considered that a dorsal approach could be efficient as a standardized procedure for LPRS [5]. The main point of the dorsal approach was that the caudate lobe was transected firstly, and the RHV could be exposed from its root to the peripheral branches and the liver parenchyma was transected from the dorsal side to ventral side (Fig. 5B). Besides, a venous cranioventral approach, exposing RHV from the root and initially transecting parenchyma at the ventral side, was also reported to be a convenient procedure [19, 20]. With a certain benefit, these described surgical methods provided alternatives for LRPS. Based on our experience in laparoscopic liver resection, LRPS was designed using the caudodorsal approach, exposing RHV from the periphery and transecting parenchyma from the dorsal side (Fig. 5C). In contrast with previously reported procedures, the present method had the following advantages.

Through the caudodorsal approach, the main trunk of the RHV could be easily exposed with only a small amount of parenchyma dissection, as a result of the thinner parenchyma at the dorsal side. With the guidance of the RHV, the liver parenchyma at the ventral side was rapidly transected, which improved the efficiency of liver dissection. Also, the branches of the RHV draining the right posterior section could be exposed and cut earlier, conducing to decreased blood loss of the remaining parenchyma dissection. Besides, the root of the RHV located deep and the surrounding space for maneuverability was limited. By the dorsal combined with caudate lobe-first approach, the operation would be affected in case of excessive bleeding, especially when the exposure was insufficient during the dissociation. Compared with the dorsal approach, exposing from the periphery by the caudodorsal approach was considered easier to master and probably more in line with the practice of most surgeons. Furthermore, it should be kept as smooth as possible for the venous return to reduce congestion in the section draining into the vein. For LRPS with the caudal approach, the right lobe usually had to be adequately mobilized and lifted up, and thus the RHV would be greatly deviated from the inherent anatomical position, leading to obstruction of venous return and an increased risk of bleeding. While during our practice using the caudodorsal approach with in situ split for LRPS, the RHV could be kept in its original position to the most extent. This was attributed to that the posterior section was in situ and the anterior section was pulled to the cranoventral side during the split, achieving a nearly horizontal dissection plane. Consequently, the venous return from the RHV was validly guaranteed. More importantly, it was relatively difficult for dissociation of the perihepatic ligaments and mobilization of the right liver due to the deep location. Our method completely avoided the problem and followed the ‘no-touch’ principle in malignancy operations, lowering the risk of tumor cells dissemination caused by intraoperative compression [21, 22].

According to other literatures about LRPS, this method did not lead to more blood loss or longer operation time [5, 10, 23, 24]. It should be noted that the postoperative length of stay was higher because most patients required the wound sutures to be removed before discharge (usually 10–12 days after operation). Overall, the intraoperative and postoperative results were satisfactory for the 11 patients who underwent this novel surgical method for LRPS. Attention should be paid to the difference in anatomic perspective of the RHV and its tributaries due to the nearly horizontal plane. Considering the difficulty to identify the diaphragmatic demarcation under the limited view, ICG fluorescence imaging was recommended by our method. As important advances in laparoscopic liver surgery, laparoscopic ultrasonography and ICG fluorescence imaging prevent the problems of tactile free and difficulty in accurately maintaining the cutting plane in laparoscopic hepatectomy [25]. Our center had gradually applied these techniques to laparoscopic hepatectomy as a routine, mainly for the inspection of small lesions, the determination of the cutting plane, and the guidance of tumor ablation.

This study had some limitations including fewer cases and the lack of a control group. Besides, outcomes in higher BMI (35+) patients via this approach were unknown yet and need further investigation. We will continue to explore the application of this method in LRPS and other laparoscopic hepatectomies, hoping to provide ideas for the standardization.

## Conclusion

The preliminary clinical effect of the caudodorsal approach with in situ split for LRPS was satisfactory. Our method was feasible and expected to provide ideas for the standardization of LRPS. Further researches are required due to some limitations of this study.

## Supplementary Information

Below is the link to the electronic supplementary material.

Supplementary file1 (MP4 329294 KB)

Supplementary file2 (MP4 362314 KB)

Supplementary file3 (MP4 291705 KB)
